# Studying Health Inequalities in Colombia: Diverse Perspectives on Their Causes and Next Steps for Action

**DOI:** 10.3390/ijerph23030297

**Published:** 2026-02-27

**Authors:** Lucinda Cash-Gibson

**Affiliations:** 1 JHU-UPF Public Policy Center (JHU-UPF PPC), UPF-Barcelona School of Management (UPF-BSM), Universitat Pompeu Fabra (UPF), 08002 Barcelona, Spain; lucinda.cash@bsm.upf.edu; 2 Department of Management, Law, Society & Humanities, UPF-Barcelona School of Management (UPF-BSM), 08008 Barcelona, Spain; 3 Research Group on Health Inequalities, Environment, Employment Conditions Knowledge Network, Department of Political and Social Sciences, Universitat Pompeu Fabra (UPF), 08002 Barcelona, Spain

**Keywords:** Colombia, health inequalities, health equity, research production, history, politics, social medicine, Latin America

## Abstract

**Highlights:**

**Public health relevance—How does this work relate to a public health issue?**

- Understanding and reducing health inequalities—systematic differences in health outcomes amongst different social groups in a population—are of critical importance for public health.
- Context-specific insights into how health inequalities are understood and studied are essential to inform future research and action.

**Public health significance—Why is this work of significance to public health?**

- The historical and global study of health inequalities includes diverse philosophical, conceptual and methodological debates on their causes, the approaches to study them, and how and where to intervene. However, not all these perspectives are widely recognized or known worldwide.
- Study findings offer valuable context-specific insights into the field of health inequalities in Colombia, including the diverse perspectives and experiences within the national research field, which not only reflect conceptual differences, but also different ethical, ontological, political proposals on how society should be organized and transformed to reduce these inequalities.

**Public health implications—What are the key implications or messages for practitioners, policy makers and/or researchers in public health?**

- Findings emphasize the importance of considering historical, political and social context that generate and exacerbate health inequalities in particular settings when trying to understand causality, make political decisions, and develop interventions.
- Findings include relevant ideas on how to create meaningful change towards building a fairer society in Colombia and beyond.

**Abstract:**

The historical and global study of health inequalities includes diverse philosophical, conceptual and methodological debates on their causes, how best to study them, and how and where to intervene. However, not all the perspectives in the field are widely recognized or known worldwide; for instance, Latin America’s long-standing intellectual tradition on Social Medicine and Collective Health. Also, evidence suggests that not all countries have had the same socio-politics and institutional conditions to enable them to produce this type of research, and a few scholars have called for more in-depth understanding of the health inequalities research field in different global settings. This study aims to assess these issues in Colombia, where the Latin America Social Medicine and Collective Health tradition remains strong, yet the volume of scientific output has not been consistent over time. In total, 12 semi-structured interviews were conducted with key informants who have worked on health inequalities in Colombia over the past five decades to understand their perspectives on these issues and the national health inequalities research field. Data was triangulated with gray and scientific literature. The findings provide valuable context-specific empirical insights into the causes of health inequalities in Colombia, the main perspectives used to study them in the country, including the associated tensions around causality, as well as ideas on how to create meaningful change towards building a fairer society.

## 1. Introduction

Since the 19th century, researchers and public health reformers have tried to understand how social conditions affected health and disease, and why health inequalities exist [1,2,3,4]. Within the historical and global study of health inequalities, there have been long-standing philosophical and theoretical debates over the approaches used to understand their main causes and trends, and where and how best to intervene to tackle them [2,5,6,7]. However, not all these perspectives in the field are necessarily widely known or appreciated globally; for instance, Latin America’s long-standing intellectual tradition on Social Medicine and Collective Health (LASM-CH) [8,9,10]. In addition, evidence suggests that some countries in the region have had more favorable socio-politics and institutional conditions to conduct this type of research than others [8,9,11], and that different perspectives on health inequalities exist within and across countries in the region [12,13]. This paper aims to explore the field of health inequalities in Colombia, where the LASM-CH tradition remains strong.

Social medicine has been a widely respected field of research and teaching in Latin American for the past several decades. The LASM-CH research tradition and movement emerged in reaction to authoritarian regimes through the 1970s and 1980s [9], and evolved in response to particularly historical, political, and economic developments and social struggles for justice and dignity throughout the region [13,14], with several key organizations and institutions founded [8,9,15]. There are ties to 19th century European social medicine, particularly Rudolf Virchow’s work, which concluded the main causes of diseases were ‘pathological and political’ in nature; therefore, the solutions required fundamental social and political change [1,11]. Followers of Virchow’s work apparently immigrated to Latin America, where social medicine perspectives interacted with Marxist perspectives in Latin American social theory [15,16,17]. LASM-CH perspectives “*…envision populations, as well as, social institutions, as totalities whose characteristics transcend those of individuals…* [and] …*conceptualises* ‘*health-illness*’ *as a dialectic process*, *rather than a dichotomous category…*” [9] (p. 1594). A strong focus is placed on how society is organized, and on the historical and (geo-) political roots of illness and health inequalities, going beyond genetic or behavioral factors, risk-factor logic and biomedical models of health that individualize and depolarize disease and inequality [8,13,18,19]. This includes analysis of the inherent power relations tied to the expansion of capitalism, colonialism and international dependency, neoliberalism and neoliberal globalization, which promoted the privatization commodification of public services, weakening of social safety nets, increased social inequality and shaped the unequal distribution of health and morbidity. There is also a strong commitment to praxis [12,13,15,18,19,20].

In terms of health inequalities’ scientific output in the region, Almeida-Filho et al. [21] analyzed the research field in Latin America and the Caribbean from 1977 to 2000, and identified three main regional ‘epicenters’ in Brazil, Mexico and Chile, with other countries’ volume of health inequalities’ scientific output being less consistent over time.

More recently, Cash-Gibson et al. [22] analyzed the historical and global health inequalities’ research field (1966–2015) and found the same regional ‘epicenters’ and suggested that they were in line with what other scholars stated about how some countries in the region at the time had more favorable institutional conditions for social medicine research (i.e., in Mexico, Ecuador, Brazil and Cuba) than others (i.e., in Argentina, Chile and Colombia) [5,9,11,21,22]. Cash-Gibson et al. [22] also found Colombia’s volume of health inequalities’ scientific output had increased significantly in the 15 years post Almeida-Filho et al.’s regional analysis [21]. These findings pose interesting questions about the contextual conditions that enable or inhibit the production of health inequalities research in different countries over time. Few descriptive analyses of the research field in Colombia, Brazil and Mexico have been conducted, and only within a few year periods [12,23,24]. However, more in-depth historical analyses of the national research fields are warranted, particularly in Colombia where the volume of scientific output has been found to vary over time. This is in line with several scholars who have called for a more in-depth understanding of the health inequalities’ research production process in different countries worldwide [5,21,25].

## 2. Materials and Methods

This study forms part of a larger case study focused on exploring Colombia’s health inequalities’ research production process and research capacities over the past five decades. In total, 12 semi-structured interviews were conducted with key informants until thematic saturation, and the data were triangulated with gray and scientific literature [26].

Potential study participants were selected based on the following inclusion criteria: (i) a researcher working on health inequalities and social medicine in Colombia, of any gender; and (ii) have produced research on health inequalities and social medicine in Colombia during the past five decades. Participants were identifiable from the bibliometric analysis of the research field [22], as well as via snowballing techniques (interviewees will be asked to suggest names of other people to contact). Potential participants were contacted via email and asked if they would like to participate in this project via an interview and sent the list of interview questions.

In terms of study participant’s profiles, the majority were Colombian (*n* = 10/12), two were foreign but had experience of working in the research field in the country based in an academic institution in the country at present (*n* = 9/12), and female (*n* = 7/12). They had a range of professional and disciplinary training, including first degrees in Medicine (*n* = 6/12), Dentistry (*n* = 3/12), Biology (*n* = 1/12), Public Health (*n* = 1/12), as well as additional postgraduate training in Epidemiologist (clinical, or social) (*n* = 6/12), Public Health (*n* = 9/12), Economics (*n* = 2/12), Education (*n* = 1/12), and Sociology (*n* = 1/12). Most of the participants were trained in several disciplines. One participant was an Emeritus Professor, one was an early career researcher, and the rest were mid to late career researchers and public health professionals (*n* = 10/12).

Participants signed an informed consent form prior to their interview in line with ethics approval. Interviews were conducted online in Spanish by the author. All interviews were recorded and conducted in line with ethics committee approval (Ethics approval received 31 October 2023 IRB ID UPFBSMAPR00012023) and transcribed from Spanish into English. All data were anonymized by the removal of any personal information that might reveal their personal identity. Participants were coded as P1–P12 in the results. The original and anonymized data were stored separately in secure external hard drives. Data was initially coded using Microsoft Word and analyzed all the texts to identify recurrent themes, which were reviewed several times. Interview data were triangulated with gray and scientific literature published in English and Spanish to help cross-check evidence and strengthen the validity and credibility of the findings. Generative artificial intelligence has not been used to develop this paper.

## 3. Results

The results have been organized into the following sub-sections: context-specific determinants of health inequalities; perspectives, tensions, and how best to study health inequalities; and how to create meaningful change.

### 3.1. Context-Specific Determinants of Health Inequalities

Compelling evidence demonstrates how the unique historical, social and political context has severely impacted Colombian society at many levels [8,9,11,13,18,27]. For several decades, Colombia has experienced intense armed conflict, involving drug cartels, military forces, paramilitary groups, and revolutionary organizations, which reached an intense level during the 1980s which has negatively affected the health and quality of life of Colombians [8,16,27]. The Colombian State attempted to negotiate for peace several times, without success; however, in 2012, a new attempt for peace was made with the guerrilla group, Revolutionary Armed Forces of Colombia-People’s Army (FARC-EP), to seek a negotiated solution to the on-going conflict. The peace agreement process took nearly four years of negotiations, during which there were many demonstrations and signs of the will for peace to solve political disagreements, and were successfully concluded in 2016 [27]. The following quote helps to illustrate how these contextual conditions have deeply impacted the country and are linked to health inequalities:

*“…we have a very complex reality…we are one of the countries with the greatest inequality in the Latin American context, and Latin America is one of the most unequal regions in the world. It is a reality that, let’s say even though economic indicators have improved…the levels of poverty, the levels of inequality continue to be very high … I believe this has a lot to do directly with the origin and with the deep causes of the long armed and social conflict that our country has lived through for more than 60 years* … *social mobilization in Colombia has been strongly marked by the war and the armed conflict…*”. *P6, Professor.*

*“…an element that has influenced all these years of internal armed conflict, which has many roots… is this immense social and socioeconomic inequality and that was not talked about so openly because of fear… we have not overcome it completely yet, but we are moving towards overcoming the conflict…” P5, Professor.*

Additional evidence suggests that during these decades of conflict, many LASM-CH advocates, because of their work in local communities and attempts to promote social reform, were targets of violence and many had to go into exile [15,16,17]. Several participants explained how these contextual conditions have limited the possibilities to discuss, analyze, or act on health inequalities; however, the more recent perception is that the peace agreements have helped create more enabling conditions to allow these issues to begin to be discussed more openly, as the following quotes illustrate:

*“…in Colombia there is an extremely critical issue, insecurity… people who are trying to fight from their communities, making changes or denouncing situations of inequality or situations of violence that perpetuate this inequality. When the processes of talking about these things, and denouncing and bringing these complexities to public light begin to take shape, these leaders are persecuted, threatened, even murdered to silence them… this continues to be very tenacious…there are groups that are still armed…paramilitary groups are the ones who control a community of displaced people…and do not allow the civilian population to organize itself, to transform…I think that this fosters more inequality because it takes away opportunities…these aspects of violence and armed conflict must be considered in the study of inequalities…it is a very particular reality of ours…” P2, Professor.*

*“…I think the war limits everything. It limits any possibility of thinking, because of the fear…and the violence that was experienced in academia…Before, it was fear that silenced people. I think that now, well, we are still afraid in many contexts, and we still feel vulnerable to the possibility of a resurgence of war, but I believe that the things that the country has done for peace have allowed us to talk about inequality…” P11, Researcher.*

*“…the peace process with the FARC has helped…at the heart of the social demands of this mobilization is the discussion on social inequalities… the peace agreement opened possibilities for discussion, for social expression, for political expression…”. P6, Professor.*

There was considerable consensus on these issues, while there was less consensus on how best to study health inequalities, which has practical as well as research implications.

### 3.2. Perspectives, Tensions, and How Best to Study Health Inequalities

Study findings suggest that diverse philosophical perspectives on the causes of health inequalities are prevalent within the country, in line with different ideologies and values. The following quote helps to explain this:

*“…there are also perspectives that make a clearer distinction between one thing is science and another thing is politics… I think that in Colombia, the reflection, the work on inequalities… has been very much influenced by a fundamental discussion in the country on the causes of inequalities and different ideological approaches and value systems to judge this*.” *P6, Professor.*

Since the WHO Commission on the Social Determinants of Health (SDH)’s 2008 report, the SDH perspective has gained international recognition and momentum in the health inequalities field [28,29]. The SDH perspective considers upstream structural factors (e.g., public policies, labor market, sociocultural values, etc.) that can shape and interact with the downstream determinants (e.g., lifestyle choices, individual behaviors, etc.) to produce health inequalities. Despite some similarities to the LASH-CM perspectives, several scholars, particularly LASM-CM scholars, critiqued the WHO’s Commission on SDH perspective. The critiques focused on its limited historical and political perspectives on the causes, and limited assessment of these living conditions and determinants, stating that it fails to consider how to address the underlying geopolitical forces and social mechanisms that exploit, oppress, dominate, discriminate and generate health inequalities [8,13,20,30,31]. In addition, these critiques considered the SDH perspective to privilege high-income countries and Anglo-Saxon experiences and approaches, downplaying the relevance of other significant scientific perspectives in the mainstream literature [13,17,30]. The following quotes illustrate some of the perceived differences between these research perspectives, as well as some of the tensions associated with their use within the health inequalities research field in Colombia:

*“…to talk about social inequalities in health, it is an ethical and political discussion…there are certain groups that make this explicit especially those who work more in the field of social determination, they make their political, ethical-political position more explicit …having rather different approaches to the conceptualization of social justice, to the conceptualization of equity in health itself, can lead to different judgements and different actions in the face of the same metric… a large part of the academic production on social inequalities involves measuring the metrics of inequalities, social inequalities, then there is no express and explicit manifestation of an ethical, political, moral, ideological position regarding what is being developed, but rather…as if it were a neutral field of discussion…” P8, Professor.*

*“…the research comes from two perspectives, which is also part of the polarization that you see right now in Colombia, the perspective of the people who are against the system, and the perspective of the people who are in favor of the system…the neoliberal system and how it is working in the country at this moment, but also about the health system…So, two poles are generated… those who speak in favor of better education, better health resources for all, that is to say, the Castro-Cuba, and Chavez-Venezuela type…and then the other pole, which is the one that wants the current system to continue working…there is a discussion about what ’social determination [of health]’ is and what ’social determinants [of health]’ are…” P4, Professor.*

The Social Determination of Health approach is heavily influenced by LASM-CH perspectives and critical social sciences; it places focus on the underlying historical, political, and economic processes and systems of exploitation and domination that generate unequal health outcomes in society and includes praxis. Structural transformation, political activism, and community engagement—particularly relevant in Colombia—are advocated for addressing health inequalities, where affected communities actively collaborate in identifying problems and finding solutions to transform the determinants that affect them [12,13,15,18,20,32]. Several study participants who use LASM-CM and Social Determination of Health perspectives discussed the main tenets, as exemplified in the following quotes:

“*…our perspective is a public health perspective… but theoretically it explains it from a social framework what produces [health inequalities], and that this social framework is a mesh, woven from the political, economic and cultural order and this…has required us to make connections with readings that are not exclusively in the field of health…the disciplines of political science and political ecology”*. *P6, Professor.*

*“…you can’t do social medicine and social determination, including inequality and equity, among other things, without a praxis that unites theory and action…one of the assumptions of what it means to do effective work in this area, that in one’s day-to-day praxis, this is intellectual and scientific work that’s intimately interrelated with political practice…” P10 Emeritus Professor.*

A few participants who use the SDH perspective in their research mentioned how they have encountered challenges with other colleagues who use other perspectives, such as the Social Determination of Health. These types of tensions may also have practical implications, as the following quote suggests:

*“I am involved in the perspective of social determinants of health…I am an epidemiologist, but those in Latin American Social Medicine talk about critical epidemiology, and really what they do is criticize epidemiology, they consider that we…help to reduce the problems that are being generated, in order to try to overshadow the bad things that the system is doing, harming people…so, we are the bad guys because we are on the side of the capitalists or neoliberals who are favoring the system…I have had this discussion with people who are in the field of social determination and I have told them, “okay, we have the same problem. We have an affected population, and we want to have a better result for that population…we must make a proposal in the middle…the problem is that social determination seeks giant transformation solutions, that is, structural transformation…of course, there must be some structural change at some point, but in the meantime, why are we going to let these populations continue to suffer and die?” P4, Professor*.

In addition, other participants consider these different perspectives to be complementary and equally necessary to advance understanding and effective action on health inequalities, as the following quotes illustrate:

“*…some, due to their training and their ideology, as well as developing qualitative research, seek to answer other types of questions, while other groups are more of a quantitative nature showing inequalities from another perspective, in another way, but I think that the two have been complementary, and there is very valuable research from both perspectives.” P1, Professor.*

“…*the critical view is healthy…but some groups that work on these issues are so much of the opinion that everything comes from political, social, history that any illness is caused by this…poverty causes all diseases…we also have biology, and biology also sometimes works against those of us who move less, who eat less, all of this, so sometimes ideology forgets the other side, and I think the balance is important.” P7, Professor.*

These findings suggest that the research field on health inequalities is divided within the country, which likely has implications for the type of health inequalities research output, research networks formed, and decisions made about how and where to best intervene to tackle these inequalities.

### 3.3. How to Create Meaningful Change

Several participants who used and advocated for the different perspectives also shared their ideas on the potential ways to advance action on health inequalities. The need to create transdisciplinary collaborations and foster creative dialogs was noted, as well as the need to actively include affected communities in identifying factors that negatively affect their health, and in searching for alternative ways forward to transform them to create fairer, healthier societies. The following quotes illustrate some of the ideas for change:

***“*** *I think that we will have to incorporate other actors into the arena…new proposals will have to be generated…but for that we do need to include other disciplines…” P4, Professor.*

“*…there is also very invisible work…especially in terms of social leadership…where, based on very particular situations in the territories, people begin to work on elements that are undoubtedly linked to inequalities and how to overcome them, which are never documented…and the interest is not guided by academic prestige, it is to solve the real issues of the territories, the real problems…[we must] connect more with the territories and the people, to get out of the academic bubble a little and make greater contact with reality…to contribute to these transformations concretely…it would be worth starting… a dialogue between the academic world and the popular world…to be able to give value to scientific knowledge in everyday life, in social dynamics… and…art is a possible nexus, a bridge…where we all feel, let’s say on an equal footing… it allows that conversation to flow much better…”. P8, Professor.*

*“… [we] must have an ideology that motivates us precisely to generate transformation, to ensure that there is an important social appropriation of knowledge with a small transformation of the reality of these societies…we need more citizen awareness, we also need more social participation, in other words, that you as a citizen feel that you can manage processes of change, even on a small scale, that you don’t have to accept everything as it is…” P2, Professor.*

Evidence suggests that there is a rich academic debate in the country about how to effectively study health inequalities and how to best act to reduce them. Yet, despite the differences, some of these ideas could be used in combination to try to establish effective ways of working towards achieving social justice in the short, medium and long term.

## 4. Discussion

Colombia’s extensive history of armed conflict, alongside persistent violence and growing inequalities, has had, and continues to have, a substantial and multifaceted negative impact on the health and well-being of its population [27]. This study’s findings offer valuable context-specific insights into the field of health inequalities in Colombia over time, including diverse perspectives and experiences in the national research field. As some scholars have explained, the global debates shaping the health inequalities agenda do not only reflect conceptual differences, but different ethical, political proposals on how society should be organized and transformed to reduce these inequalities [2,5,13]. However, not all these perspectives and proposals are widely known. This study’s findings demonstrate how these types of international debates also occur within countries, particularly in Colombia, despite wide-spread fear. Some evidence suggests that the tensions that lie within the national research field are linked to diverse disciplinary training, ideologies, values, and ontological assumptions. These findings are also in line with findings from other countries in the European region [25], which highlighted the “*strong political nature*” of the national health inequalities’ research production process (p. 2). Similarly to Colombia, these countries had “*…strong traditions of public health and recognition of social justice*, [and]… *individual egalitarian values and ideologies [were found to] have played a significant role in initiating and sustaining the production of [this] research, which likely occurred* via *the activation of a sense of moral responsibility to act (mechanism) and to prove that of [health inequalities] existed and to try to address them”* [25] (p. 2–3). In addition, evidence suggested that these contextual factors and mechanisms were *“…particularly important during periods of struggles or in “hostile socio-political and research environments*…*to keeping the research field alive, and likely influenced the type of research produced* (e.g., *the psychosocial explanations versus the social-material conditions*)” [25] (p. 2-4). These findings also resonate with the Colombian context.

Debates over the causes of health inequalities and how and where to intervene to tackle them are of importance, as “…*rigorous questioning is the engine of transformation*” (p. 7) [33]. It is also important that researchers and policy makers are clear about the implicit and explicit theoretical and ontological positions embedded within certain perspectives when using them to guide proposals for social change [34].

LASM-CH perspectives advocate structural and political change and transformative processes and collective action to address the deep roots of these inequalities. These include the involvement of social movements, community participation in health decision-making processes, and building collective power to advocate for changes in public policies, economic models, and social structures that impact health to push for structural reforms to act on the root causes of health inequalities [13]. Some evidence also suggests that creative, artistic and transdisciplinary practices should be put in place to foster recognition and identification of the problems, build a comprehensive knowledge base built on integrated perspectives and experiences from different actors, and mobilize collective action aimed at challenging the structural drivers of health inequalities in the country. Several scholars, within and outside the country and the Latin American region, consider participation to be key for transformative social change, as “*…it offers an alternative to the prevailing views of society…to find this new paradigm will require compromise. While there are substantive differences of views between people and vested interests on the left and right that will resist change*” [35] (p. 103–106).

In addition, evidence from a recent cross-country case analysis (i.e., Brazil, The United States, United Kingdom, and Germany) identified a few mechanisms that enabled these four countries to reduce health inequalities during different historical periods [36]. Namely, a combination of macro-level policies aiming to address social inequalities more broadly are found to be required to reduce health inequalities in these countries, including the welfare state expansion, improved access to quality healthcare, reduced income inequality, and enhanced democratic participation [36]. Also, they found that social movements helped to push for progressive policies which have helped build political will to enact these reforms [36]. Other potential levelling mechanisms, related to housing, land ownership, and addressing the commercial determinants, were mentioned as warranting further investigation. Many of these factors also seem relevant for the Colombian context and are in line with what LASM-CH perspectives advocate need be done to try to reduce health inequalities in the region.

Furthermore, similarly to what one study participant mentioned, other scholars have recently mentioned how art “…*can serve as a powerful medium for triggering and contextualizing imaginaries, as it enables participants across diverse geographies, knowledge systems, governance levels and socio-demographic backgrounds to engage meaningfully with [a] topic and express their visions of a future…[serving as]… a means to channel emergent and more relational thinking around complex [environmental and social] challenges by helping people connect with emotions and reveal underlying tensions*…” [37] (p. 1223). This is important as it helps to expand our understanding of social reality, beyond what is (made) ‘visible’ in academic (internationally), therefore expanding the current horizon of possibilities for social action and positive transformation [5,38]. Collectively, this may also help to rebuild our moral imagination, that is, our individual and collective ability to empathize with one another and create a fairer society [35,39]. Future research and action should explore these ideas further.

In terms of strengths, this study provides new empirical evidence on the diverse perspectives within the health inequalities research field in Colombia and builds on previous studies that explained the different conceptual perspectives prevalent in the country and region [10,12,13,20]. In terms of limitations, as this study stemmed from a wider investigation/case study focused on understanding Colombia’s health inequalities’ research production process and related capacities over time, only academics were interviewed. Further research should therefore explore the perspectives and roles of other stakeholders working to understand and address health inequalities in the country. This could enrich learning and help to consider alternative ways of creating meaningful change towards reducing health inequalities at the local and global level.

## 5. Conclusions

Colombia is faced with a very complex reality, which includes a long-standing history of violence and huge social inequalities. The country also has a tradition of conducting critical social research and studying health inequalities. Historically, these insights have not been widely shared or read outside the region of Spanish-speaking parts of the world; however, important efforts have translated major works from Spanish into English to increase their visibility internationally. Further efforts are still needed to ensure that this type of work is spoken about and continues to be made visible, both nationally and internationally.

This study provides valuable empirical insights into the diverse academic perspectives on health inequalities in Colombia, including some of the dynamics and tensions within the national research field, and ideas on what could be done to create necessary changes and support action on these inequalities. In line with previous research, these findings emphasize the importance of considering historical, political and social contexts that generate and exacerbate health inequalities in specific settings in both research and practice.

Creating alternative imaginaries and proposals for the future and building meaningful change towards a fairer society—in Colombia and other countries worldwide—requires extensive efforts. To support these effects, comprehensive assessments of the historical, political and structural determinants of health inequalities and associated mechanisms per context are needed, as well as inclusive, creative, transdisciplinary approaches in knowledge generation and decision-making processes that go beyond existing disciplinary silos, academic/techno fixes, and dominant high-income country and Anglo-Saxon speaking perspectives, in addition to considerable negotiations.

## Data Availability

Original data is unavailable due to privacy or ethical restrictions.

## Abbreviations

The following abbreviations are used in this manuscript:

LASM-CH	Latin America Social Medicine and Collective Health
SDH	Social Determinants of Health
